# Horizontal Gene Transfer and Loss of Serotype-Specific Genes in Listeria monocytogenes Can Lead to Incorrect Serotype Designations with a Commonly-Employed Molecular Serotyping Scheme

**DOI:** 10.1128/spectrum.02745-22

**Published:** 2022-12-06

**Authors:** Phillip Brown, Zuzana Kucerova, Lisa Gorski, Yi Chen, Mirena Ivanova, Pimlapas Leekitcharoenphon, Cameron Parsons, Jeffrey Niedermeyer, James Jackson, Sophia Kathariou

**Affiliations:** a Department of Plant and Microbial Biology, North Carolina State University, Raleigh, North Carolina, USA; b Centers for Disease Control and Prevention (CDC), EDLB/DFWED, Atlanta, Georgia, USA; c U.S. Department of Agriculture, Agricultural Research Service, Western Regional Research Center, Produce Safety and Microbiology Unit, Albany, California, USA; d Division of Microbiology, Center for Food Safety and Applied Nutrition, Food and Drug Administration, College Park, Maryland, USA; e Research Group for Genomic Epidemiology, National Food Institute, Technical University of Denmark, Lyngby, Denmark; f Department of Food, Bioprocessing and Nutrition Sciences, North Carolina State University, Raleigh, North Carolina, USA; University of Adelaide

**Keywords:** *Listeria monocytogenes*, PCR, serotype, foodborne pathogens, horizontal gene transfer

## Abstract

Listeria monocytogenes is a Gram-positive, facultative intracellular foodborne pathogen capable of causing severe, invasive illness (listeriosis). Three serotypes, 1/2a, 1/2b, and 4b, are leading contributors to human listeriosis, with 4b including the major hypervirulent clones. The multiplex PCR scheme developed by Doumith and collaborators employs primers targeting specific lineages (e.g., lineage II-specific *lmo0737*, lineage I-specific *LMOf2365_2059*) or serotypes (e.g., serotype 4b-specific *LMOf2365_1900*). The Doumith scheme (DS) is extensively employed for molecular serotyping of L. monocytogenes due to its high accuracy, relative ease, and affordability. However, for certain strains, the DS serotype designations are in conflict with those relying on antibody-based schemes or whole-genome sequence (WGS) analysis. In the current study, all 27 tested serotype 4b strains with sequence type 782 (ST782) within the hypervirulent clonal complex 2 (CC2) were designated 1/2b/3b using the DS. These strains lacked the serotype 4b-specific gene *LMOf2365_1900*, while retaining *LMOf2365_2059*, which, together with *prs*, yields the DS 1/2b/3b profile. Furthermore, 15 serotype 1/2a strains of four STs, mostly from water, were designated 1/2b/3b using the DS. These strains lacked the *lmo0737* cassette but harbored genomic islands with *LMOf2365_2059*, thus yielding the DS 1/2b/3b profile. Lastly, we investigated a novel, dual 1/2a-1/2b profile obtained using the DS with 21 serotype 1/2a strains of four STs harboring both the *lmo0737* cassette and genomic islands with *LMOf2365_2059*. The findings suggest that for certain strains and clones of L. monocytogenes the DS designations should be viewed with caution and complemented with alternative tools, e.g., traditional serotyping or WGS analysis.

**IMPORTANCE** Listeria monocytogenes is a foodborne pathogen responsible for severe illness (listeriosis), especially in pregnant women and their fetuses, immunocompromised individuals, and the elderly. Three serotypes, 1/2a, 1/2b, and 4b, account for most human listeriosis, with certain serotype 4b clonal complexes (CCs) overrepresented in human disease. Serotyping remains extensively employed in *Listeria* epidemiologic investigations, and a multiplex PCR-based serotyping scheme is widely used. However, the PCR gene targets can be lost or gained via horizontal gene transfer, leading to novel PCR profiles without known serotype designations or to incorrect serotype assignments. Thus, an entire serotype 4b clone of the hypervirulent CC2 would be misidentified as serotype 1/2b, and several strains of serotype 1/2a would be identified as serotype 1/2b. Such challenges are especially common in novel clones from underexplored habitats, e.g., wildlife and surface water. The findings suggest caution in application of molecular serotyping, while highlighting *Listeria*’s diversity and potential for horizontal gene transfer.

## INTRODUCTION

Listeria monocytogenes is a Gram-positive, facultative intracellular foodborne bacterial pathogen notorious for its persistence in food-processing facilities. It is the causative agent of human listeriosis, typically via the consumption of ready-to-eat (RTE) processed foods (1). Individuals at high risk include those who are immunocompromised, the elderly, pregnant women, and their fetuses. The outcomes can be severe, with manifestations such as septicemia, meningitis, stillbirths, and abortions, and the case fatality rate remains high (estimated 20 to 30%) (2, 3).

Of the 14 L. monocytogenes serotypes, the majority of human listeriosis cases can be attributed to just three, i.e., serotypes 1/2a, 1/2b, and 4b, with serotype 4b also including the leading hypervirulent clones of clonal complexes (CCs) 1, 2, 4, and 6 (4–7). L. monocytogenes consists of four genomic lineages (I, II, III, IV), with most cases of human listeriosis involving lineages I and II. Serotypes 1/2b, 3b and most strains of serotype 4b are members of lineage I, while serotypes 1/2a, 1/2c, 3a, and 3c belong to lineage II (8–10).

Serotyping is extensively used to characterize L. monocytogenes (11). Accurate serotyping of L. monocytogenes is crucial in the early stages of source-tracking efforts and provides valuable information of relevance to *Listeria* adaptations, ecology, and epidemiology. For instance, wall teichoic acid decorations that serve as receptors for *Listeria* phages vary among serotypes, impacting *Listeria* phage host ranges (12–15). Several methods have been developed to serotype L. monocytogenes, each with their advantages and disadvantages (16). The traditional serotyping method involves agglutination reactions between antisera and cell surface somatic (O) and flagellar (H) antigens. This method is accurate but time consuming, requires panels of dedicated antisera, and the results may be difficult to interpret (17). Serotype-specific antibodies have also been employed in enzyme-linked immunosorbent assay (ELISA) protocols (11).

In 2004, Doumith and collaborators reported the first and still most widely used molecular serotyping approach for L. monocytogenes, based on multiplex PCR with five primer sets targeting *prs* (*lmo0199*), found in all L. monocytogenes strains, as well as genes associated with specific lineages and serotypes (18). The latter include *lmo0737* (lineage II); *LMOf2365_2059* (ORF2819; lineage I); *LMOf2365_1900* (ORF2110; serotype 4b); and *lmo0118*, harbored by the lineage II serotypes 1/2c and 3c (18). This molecular serotyping scheme, hereafter referred to as the Doumith scheme (DS), has since become a standard in a multitude of laboratories due to its relative ease of use and low cost (11, 19), with the original paper being cited 273 times on PubMed Central (https://www.ncbi.nlm.nih.gov/pmc/) as of 21 September 2022. The recent emergence of whole-genome sequencing (WGS) has allowed the *in silico* serotyping of L. monocytogenes strains based on core genome multilocus sequence typing (cgMLST) allele clustering (20, 21), but this remains expensive and beyond the reach of many laboratories working with large numbers of isolates.

While the DS remains an excellent tool for molecular serotyping in L. monocytogenes, it is important to be aware of potential challenges. For instance, identification of serotypes 1/2c and 3c with the DS is based on the detection of *lmo0118*, but the reference strain L. monocytogenes EGD-e (serotype 1/2a) harbors *lmo0118*, thus yielding the DS profile 1/2c/3c (18). Furthermore, profiles novel to the original DS can be exhibited by certain strains, especially novel clones that were not known when the scheme was developed. Thus, certain clones of serotype 4b harbor the lineage II-specific *lmo0734*-*lmo0739* cassette, producing a novel profile designated IVb-v1 using the DS (22–24). This cassette was highly conserved (>99% nucleotide identity) between these serotype 4b clones and lineage II, suggesting horizontal gene transfer (HGT) and dissemination of the cassette harboring *lmo0737* (23). The IVb-v1 profile is found in several emerging clones implicated in listeriosis outbreaks, including sequence types (STs) 382 and 554 (25–27). Certain strains are untypeable by the DS scheme, including atypical rhamnose-negative L. monocytogenes strains of the recently identified serotype 4h (28).

During routine applications of the DS in our laboratory, we also noted that occasionally the DS serotype designations were in conflict with those based on conventional antibody-based serotyping or analysis of WGS data. Specifically, several strains of serotypes 4b or 1/2a yielded the serotype 1/2b/3b profile with the DS. Furthermore, we noted a profile which, similarly to IVb-v1, is novel to the original DS and includes dual 1/2a-1/2b amplicons (29). The objective of the current study was to further analyze these strains in order to elucidate the underlying mechanisms.

## RESULTS AND DISCUSSION

### The serotype 4b clone ST782, member of the hypervirulent CC2, yields the serotype 1/2b/3b designation using the Doumith scheme and lacks the serotype 4b-specific *LMOf2365_1900* (ORF2110).

**FIG 1:**
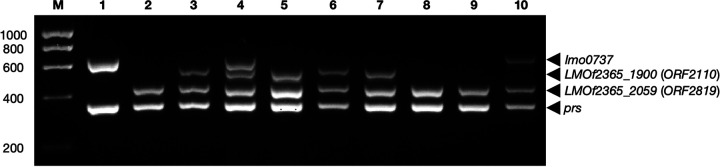
Multiplex PCR profiles of selected strains using the DS. L. monocytogenes strains are as follows: lane 1, 2010L-1723 (serotype 1/2a; ST378); lane 2, 2011L-2858 (serotype 1/2b; ST5); lane 3, F2365 (serotype 4b; ST1); lane 4, 2007-0904 (serotype 4b, DS IVb-v1; ST382); lane 5, CFSAN048783 (serotype 4b; ST2); lane 6, SKB111 (serotype 4b; ST1039, CC2); lane 7, CFSAN076297 (serotype 4b; ST2125, CC2); lane 8, PNUSAL002394 (serotype 4b, DS 1/2b/3b; ST782, CC2); lane 9, SKWL256 (serotype 1/2a, DS 1/2b/3b; ST1492); and lane 10, SKWL223 (serotype 1/2a, DS 1/2a-1/2b; ST912). Lane M, 1-kb HyperLadder molecular weight marker (Meridian Biosciences, Cincinnati, OH, USA). Base pair sizes are indicated on the left. Target genes corresponding to specific amplicons are indicated on the right. The DS was implemented as described (18). Unless otherwise indicated, the DS designations were those expected based on serotype.

**FIG 2:**
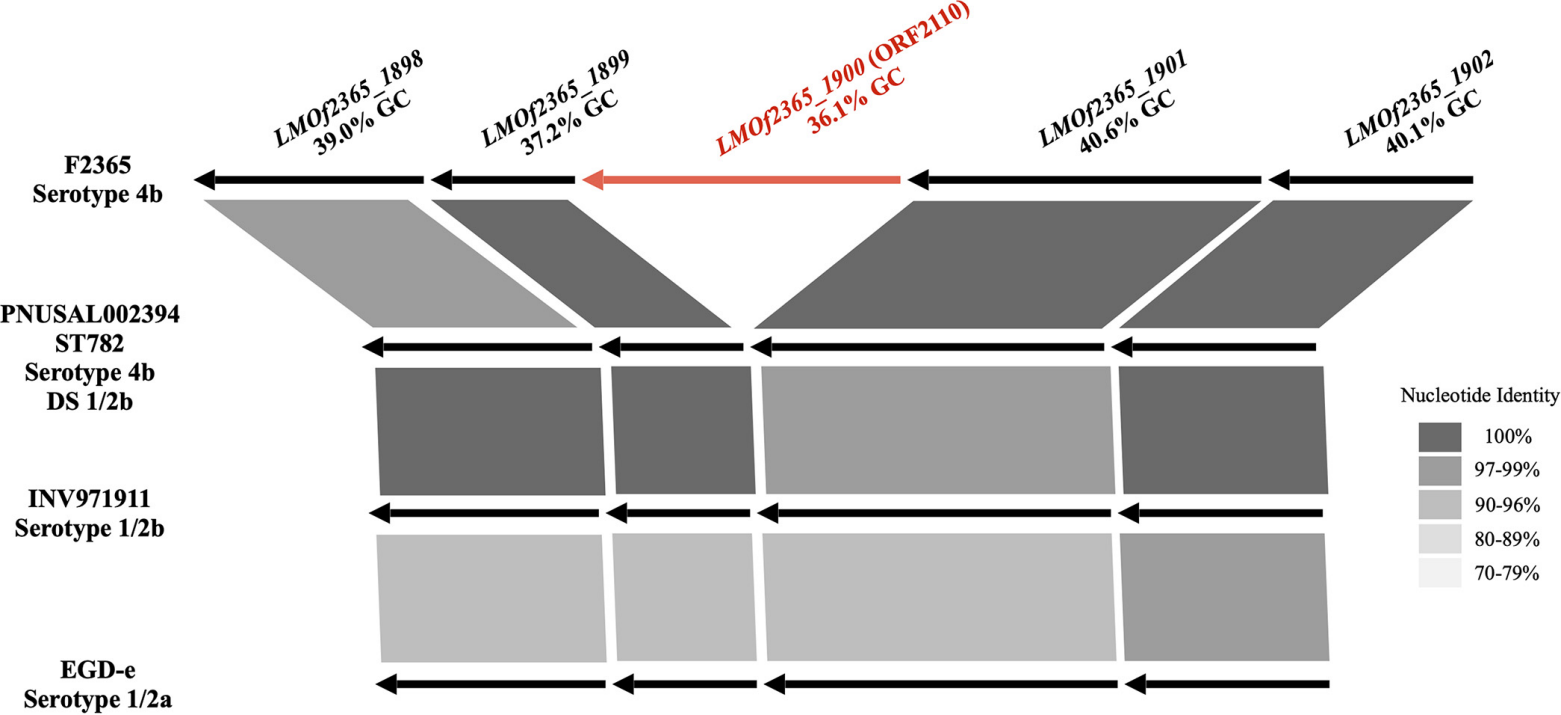
Genomic organization of *LMOf2365_1900* (ORF2110) and flanking genes in L. monocytogenes strains EGD-e (serotype 1/2a), F2365 (serotype 4b), INV971911 (serotype 1/2b), and the ST782 strain PNUSAL002394 (serotype 4b, with 1/2b/3b profile using the DS). Genes harbored by all four strains are indicated by black arrows, while the serotype 4b-specific *LMOf2365_1900*, used as the target in the DS, is in red. The nucleotide identity levels of genes between different strains are indicated by gray shading. The GC content is provided for each gene in this region. Serotype designations obtained using the DS are indicated as “DS.” Unless otherwise indicated, the DS designations were those expected based on serotype.

ST782 belongs to the hypervirulent CC2 (serotype 4b); the ST782 strains that we examined were derived from human listeriosis and surface water (Table 1). All tested ST782 strains are serotype 4b based on agglutination, ELISA, and WGS analysis but, surprisingly, were all identified as 1/2b/3b using the DS (Fig. 1). In addition to *prs* (*lmo0199*), which is shared by all serotypes, serotypes 1/2b and 4b share the lineage I-specific target *LMOf2365_2059* (ORF2819), while 4b strains harbor an additional serotype 4b-specific gene, *LMOf2365_1900* (ORF2110) (18). However, analysis of the *LMOf2365_1900* genomic region revealed that this gene was absent from all tested ST782 strains, regardless of their origin (Fig. 2). *LMOf2365_1900* is annotated as a serine protease, and in other serotype 4b strains, it is in a conserved region of the chromosome between *lmo1865* and *lmo1866*, with a GC content markedly lower (36.1%) than that of the surrounding genes (39.2%) in the reference serotype 4b strain F2365 (Fig. 2). Thus, even though we cannot exclude the possibility that *LMOf2365_1900* is an ancestral L. monocytogenes gene that was lost by clones other than those of serotype 4b (and selected serotype 4b clones, such as ST782), its GC content suggests that it was acquired by serotype 4b strains via HGT and subsequently eliminated from ST782. *In silico* analysis of 11 additional ST782 strains from the *Listeria* database at the Centers for Disease Control and Prevention (CDC), including 9 human clinical strains and 1 each from food and water, revealed that all lacked *LMOf2365_1900* and would thus yield the serotype 1/2b/3b designation using the DS (see Table S1 in the supplemental material).

**TABLE 1:** L. monocytogenes strains used in this study

Strain^*a*^	SRA accession no.	Source	Yr	ST^*b*^	CC	DS	Serotype according to:		
							WGS	Agglutination	ELISA
PNUSAL001711	SRS1068411	Clinical,^*c*^ MD, USA	2015	124	124	1/2a-1/2b	1/2a	1/2a	ND^*d*^
SKB427	SRS2772091	Black bear N110, rectal swab, NC, USA	2016	912	912	1/2a-1/2b	1/2a	1/2a	ND
SKB429	SRS15800321	Black bear N110, rectal swab, NC, USA	2016	912	912	1/2a-1/2b	1/2a	ND	ND
SKWL57	SRS8215092	Creek water, NC, USA	2016	912	912	1/2a/3a	1/2a	ND	ND
SKWL62	SRS8224581	Creek water, NC, USA	2017	912	912	1/2a/3a	1/2a	ND	ND
SKWL69	SRS8215188	Creek water, NC, USA	2018	912	912	1/2a/3a	1/2a	ND	ND
SKWL223	SRS8205717	Creek water, NC, USA	2017	912	912	1/2a-1/2b	1/2a	ND	ND
SKWL227	SRS8205573	Creek water, NC, USA	2017	912	912	1/2a-1/2b	1/2a	ND	ND
SKWL252	SRS8205721	Debris in creek water, NC, USA	2017	912	912	1/2a-1/2b	1/2a	ND	ND
SKWL337	SRS15801915	Creek water, NC, USA	2018	912	912	1/2a-1/2b	1/2a	ND	ND
SKB102	SRS15800319	Black bear N041, rectal swab, NC, USA	2014	**1365**	1365	1/2a-1/2b	1/2a	1/2a	ND
SKB128	SRS8205750	Black bear N052, feces, NC, USA	2014	**1365**	1365	1/2a-1/2b	1/2a	1/2a	ND
SKB397	SRS2772120	Black bear N095, nasal swab, NC, USA	2016	**1383**	912	1/2a-1/2b	1/2a	1/2a	ND
SKB398	SRS15800320	Black bear N095, nasal swab, NC, USA	2016	**1383**	912	1/2a-1/2b	1/2a	ND	ND
SKWL39	SRS8205560	Creek water, NC, USA	2016	**1503**	1503	1/2a-1/2b	1/2a	ND	ND
SKWL40	SRS15801914	Creek water, NC, USA	2016	**1503**	1503	1/2a-1/2b	1/2a	ND	ND
SKB110	NA^*e*^	Black bear, fecal, USA	2014	ND	ND	1/2a-1/2b	ND	1/2a	ND
JJ25	SRS15801912	Creek water, NC, USA	2021	1055	1055	1/2b/3b	1/2a	ND	ND
SKB57	SRS8205774	Black bear UGA 170, rectal swab, GA, USA	2014	1055	1055	1/2b/3b	1/2a	1/2a	ND
SKWL79	SRS8215344	Debris (metal can) in creek water, NC, USA	2016	1055	1055	1/2b/3b	1/2a	1/2a	ND
PNUSAL000427	SRS531351	Clinical, USA	2013	1082	1082	1/2b/3b	1/2a	1/2a	ND
SKWL256	SRS8215338	Creek water, NC, USA	2017	**1492**	1492	1/2b/3b	1/2a	1/2a	ND
SKWL386	SRS15801917	Creek water, NC, USA	2018	**1492**	1492	1/2b/3b	1/2a	ND	ND
SKWL388	SRS15801918	Creek water, NC, USA	2018	**1492**	1492	1/2b/3b	1/2a	ND	ND
SKWL82	SRS8215330	Debris (metal can) in creek water, NC, USA	2016	**1494**	1494	1/2b/3b	1/2a	1/2a	ND
SKWL85	SRS8215187	Debris (plastic bottle) in creek water, NC, USA	2016	**1494**	1494	1/2b/3b	1/2a	1/2a	ND
SKWL143	SRS8214724	Creek water, NC, USA	2016	**1494**	1494	1/2b/3b	1/2a	1/2a	ND
SKWL155	SRS8215079	Creek water, NC, USA	2016	**1494**	1494	1/2b/3b	1/2a	1/2a	ND
SKWL159	SRS15801913	Creek water, NC, USA	2016	**1494**	1494	1/2b/3b	1/2a	ND	ND
SKWL347	SRS15801916	Creek water, NC, USA	2018	**1494**	1494	1/2b/3b	1/2a	ND	ND
SKWL431	SRS15801919	Creek water, NC, USA	2019	**1494**	1494	1/2b/3b	1/2a	ND	ND
PNUSAL000395	SRS508797	Clinical, USA	2013	782	2	1/2b/3b	4b	4b	ND
PNUSAL001042	SRS716025	Clinical, TX, USA	2014	782	2	1/2b/3b	4b	4b	ND
PNUSAL001307	SRS837087	Clinical, MD, USA	2015	782	2	1/2b/3b	4b	4b	ND
PNUSAL001515	SRS978018	Clinical, CT, USA	2015	782	2	1/2b/3b	4b	4b	ND
PNUSAL002350	SRS1596965	Clinical, MN, USA	2016	782	2	1/2b/3b	4b	ND	ND
PNUSAL002394	SRS1618757	Clinical, TX, USA	2016	782	2	1/2b/3b	4b	ND	ND
PNUSAL002732	SRS1893124	Clinical, IN, USA	2016	782	2	1/2b/3b	4b	ND	ND
PNUSAL003280	SRS2647270	Clinical, VA, USA	2017	782	2	1/2b/3b	4b	ND	ND
PNUSAL003431	SRS2681003	Clinical, GA, USA	2017	782	2	1/2b/3b	4b	4b	ND
PNUSAL004174	SRS3644761	Clinical, CT, USA	2018	782	2	1/2b/3b	4b	ND	ND
CFSAN073893	SRS6199115	Water, Salinas River watershed, CA, USA	2012	782	2	1/2b/3b	4b	ND	4b
CFSAN076306	SRS9978550	Water, Salinas River watershed, CA, USA	2014	782	2	1/2b/3b	4b	ND	4b
CFSAN078591	SRS6197421	Water, Salinas River watershed, CA, USA	2014	782	2	1/2b/3b	4b	ND	4b
CFSAN081597	SRS6197237	Water, Salinas River watershed, CA, USA	2015	782	2	1/2b/3b	4b	ND	4b
CFSAN092770	SRS6199622	Water, Salinas River watershed, CA, USA	2016	782	2	1/2b/3b	4b	ND	4b
CFSAN093485	SRS6159691	Water, Salinas River watershed, CA, USA	2016	782	2	1/2b/3b	4b	ND	4b

a SKB in the strain name designation indicates that the strains were derived as described from black bears (Ursus americanus) in the southeastern United States (29); SKWL in the strain name designation indicates that the strains were derived from water of an urban creek or from debris submerged in creek water in North Carolina, USA, as described (30). Strains isolated from agricultural surface water of the Salinas River watershed in California, USA, were derived as described (31).

b Novel STs are indicated in bold typeface.

c Clinical, strains of human clinical origin.

d ND, not determined using the respective method.

e NA, not available, as the strain was not sequenced.

### Serotype 1/2a strains that yield the serotype 1/2b/3b profile via the DS exhibit both loss of the *lmo0734*-*lmo0739* cassette and acquisition of *LMOf2365_2059* (ORF2819) in a hypervariable chromosomal region.

**FIG 3:**
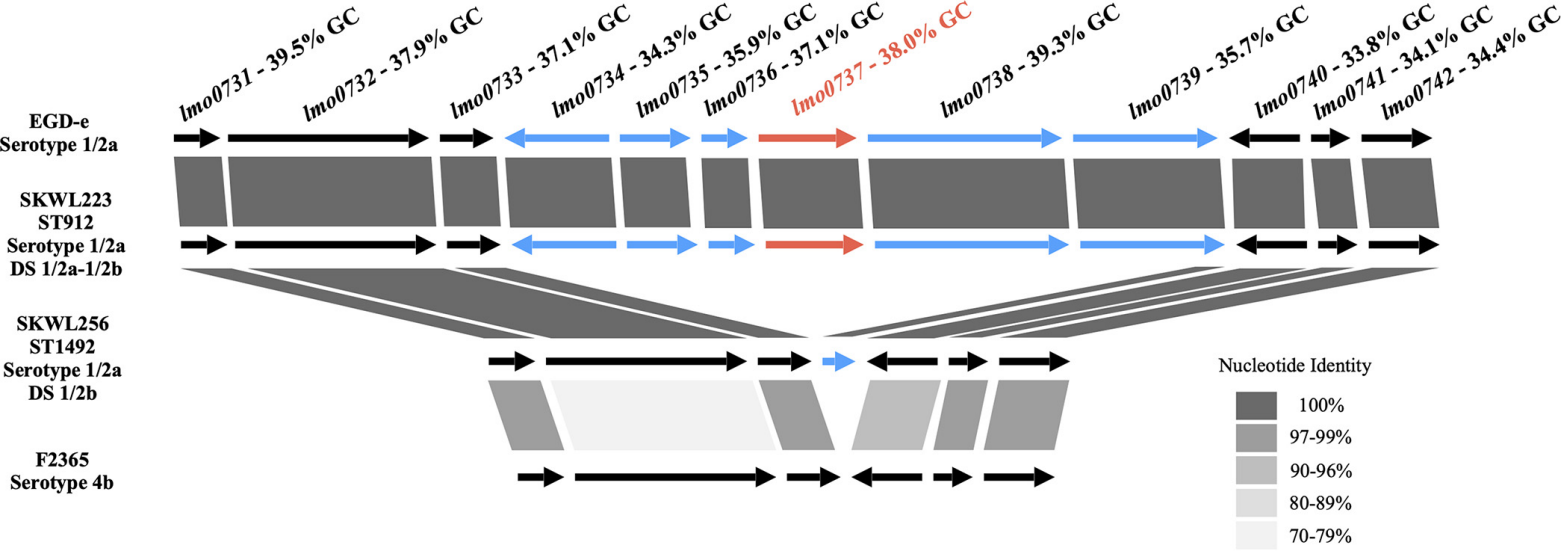
Genomic organization of *lmo0737* and surrounding genes in L. monocytogenes strains EGD-e (serotype 1/2a), F2365 (serotype 4b), SKWL233 (serotype 1/2a, with the dual 1/2a-1/2b profile using the DS) and SKWL256 (serotype 1/2a, with a 1/2b/3b profile using the DS). Genes harbored by all four strains are indicated by black arrows, while genes from the *lmo0734*-*lmo0739* cassette are indicated by blue arrows, and *lmo0737* (used as the target in the DS) is in red. Nucleotide identity levels of genes between different strains are indicated by gray shading. The GC content is provided for each gene in this region. Serotype designations obtained using the DS are indicated as “DS.” Unless otherwise indicated, the DS designations were those expected based on serotype.

Several (*n* = 14) strains of ST1055 (*n* = 3), ST1082 (*n* = 1), ST1492 (*n* = 3), and ST1494 (*n* = 7) yielded the serotype 1/2b/3b designations using the DS (Fig. 1 and data not shown), but subsequent WGS analysis and agglutination-based serotyping indicated that they were serotype 1/2a (Table 1). ST1492 and ST1495 were novel and mostly of aquatic origin (Table 1). Analysis of the *lmo0737* genomic region known to be specific to lineage II (18, 32) revealed that the *lmo0734*-*lmo0739* cassette was absent, with only a 207-bp remnant of *lmo0739* (full size, 1,374 bp) remaining, while the flanking genes remained intact (Fig. 3). Interestingly, the GC content of the cassette was heterogeneous, with some genes (e.g., *lmo0737*, *lmo0738*) having GC values similar to the L. monocytogenes chromosomal average of approximately 38% (33), while the GC content of others (e.g., *lmo0734*, *lmo0735*) was much lower (Fig. 3). Genes downstream of *lmo0739* continued to have abnormally low GC content (Fig. 3), suggesting that this entire chromosomal region may have been acquired via HGT. We also noted high diversity in the flanking genes between serotypes 1/2a and 4b. This diversity is most noted in *lmo0732*, with 77% nucleotide identity and 75% amino acid identity between EGD-e (serotype 1/2a; lineage II) and F2365 (serotype 4b; lineage I).

**FIG 4:**
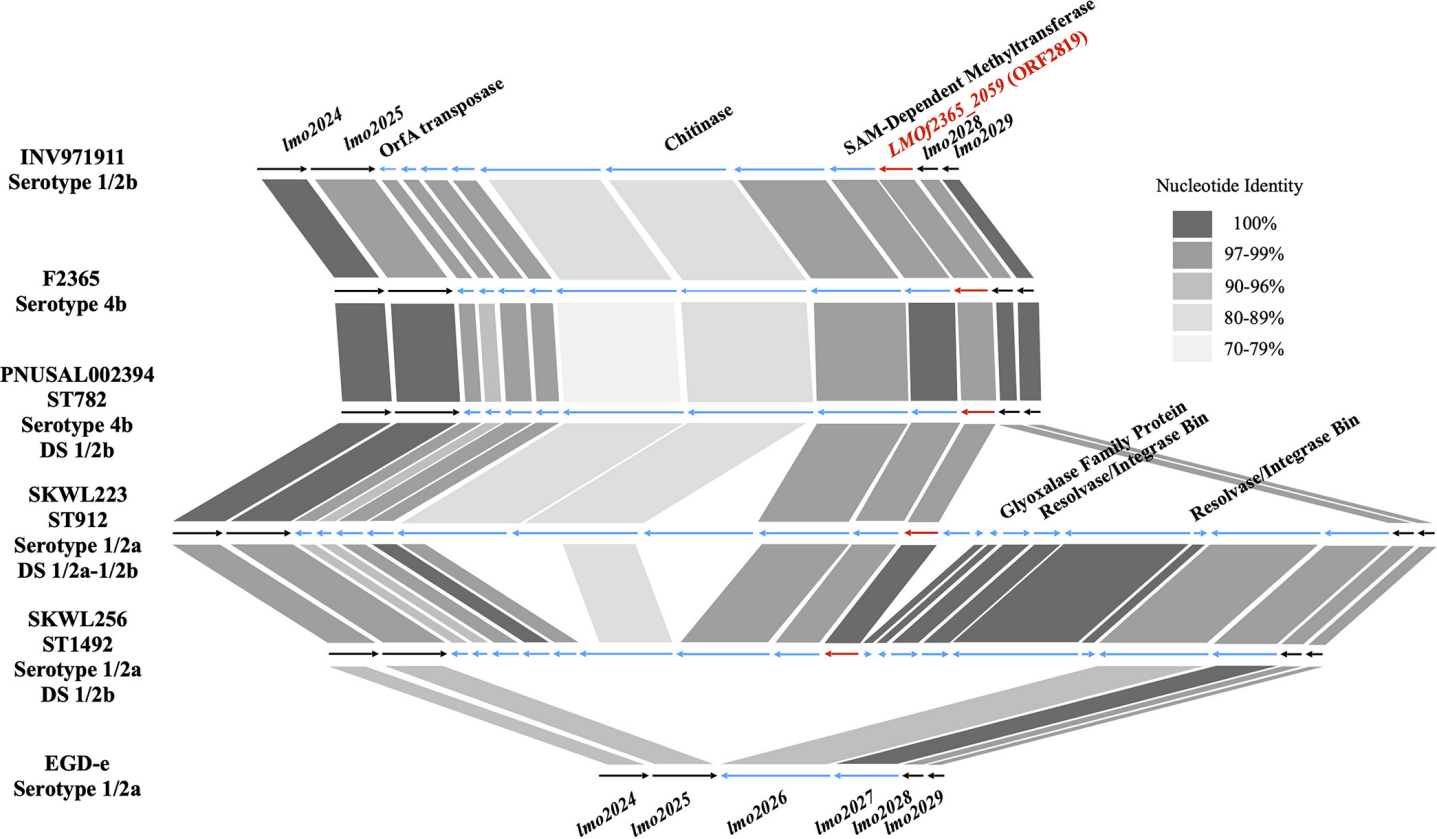
Genomic organization of *LMOf2365_2059* (ORF2819) and surrounding genes in L. monocytogenes strains EGD-e (serotype 1/2a), F2365 (serotype 4b), INV971911 (serotype 1/2b), PNUSAL002394 (ST782; serotype 4b, with a 1/2b profile in the DS), SKWL233 (serotype 1/2a, with the dual 1/2a-1/2b profile in the DS), and SKWL256 (serotype 1/2a, with a 1/2b/3b profile in the DS). Genes harbored by all six strains are indicated by black arrows, while variable genes are indicated by blue arrows, and *LMOf2365_2059* (used as the target in the DS) is shown in red. Genes were annotated using RAST, and genes without labels were annotated as hypothetical proteins. Nucleotide identity levels between genes in different strains are indicated by gray shading. Serotype designations obtained using the DS are indicated as “DS.” Unless otherwise indicated, the DS designations were those expected based on serotype.

While these strains lacked the *lmo0734*-*lmo0739* cassette, they harbored the lineage I-specific *LMOf2365_2059*, leading to a serotype 1/2b/3b designation with the DS (Fig. 4 and data not shown). Originally, we speculated that these lineage I-specific sequences may have replaced *lmo0734-lmo0739*, as was noted in other L. monocytogenes genomic hot spots (34, 35). However, we found that *lmo0734*-*lmo0739* was not replaced by novel genomic content (Fig. 3), and *LMOf2365_2059* was localized in a different genomic region, flanked by the conserved genes *lmo2025* and *lmo2028* (Fig. 4 and data not shown). The site between *lmo2025* and *lmo2028* was previously identified among major hypervariable hot spots in the L. monocytogenes chromosome (36). As will be discussed further below, in addition to *LMOf2365_2059*, the serotype 1/2a strain SKWL256 with the DS 1/2b/3b profile harbored several additional genes in this region that were absent from the genomic counterparts of either EGD-e (serotype 1/2a) or lineage I, e.g., strains F2365 and PNUSAL002394 (both of serotype 4b) or INV971911 (serotype 1/2b) (Fig. 4).

Serotype 1/2a strains yielding the 1/2b/3b profile using the DS were uncommon in our strain collection and were members of recently identified STs; as indicated earlier, ST1492 and ST1495 were novel STs first identified from water and water-immersed objects (Table 1). To determine whether the findings were specific to the corresponding STs, we interrogated the L. monocytogenes database at the CDC and the Institut Pasteur’s BIGSdb (https://bigsdb.pasteur.fr/listeria/) for additional strains with the same STs. No additional ST1492 strains could be identified, suggesting that this novel ST remains uncommon. However, we identified two additional strains of ST1055 (one each from fresh produce and blood), as well as a blood-derived strain of ST1494 and 28 strains of ST1082, mostly from food and environmental swabs (Table S1). The additional ST1055 and ST1494 strains (Table S1) lacked *lmo0737*, while harboring *LMOf2365_2059*, suggesting that this trait may be a clonal feature of these two STs. On the other hand, *in silico* screening of the additional ST1082 strains indicated that they would yield the 1/2a/3a DS designation (Table S1). Thus, the findings for the ST1082 strain in our panel (Table 1) may be specific to this strain.

### Strains with a dual 1/2a-1/2b profile with the DS have acquired a hypervariable mobile genetic element harboring *LMOf2365_2059* (ORF2819).

Certain L. monocytogenes strains from black bears were found to exhibit an unusual dual 1/2a-1/2b profile using the DS (29). Interestingly, a strain from human listeriosis and several L. monocytogenes strains from surface water also exhibited this profile (Fig. 1 and data not shown; Table 1). The strains yielding the dual 1/2a-1/2b profile using the DS harbored the lineage II-specific *lmo0734*-*lmo0739* cassette (Fig. 3 and data not shown) but also *LMOf2365_2059*, responsible for the lineage I-specific amplicon in the DS (Fig. 4 and data not shown). These strains were serotype 1/2a and members of several different STs, i.e., ST124, ST912, and the novel ST1365, ST1383, and ST1503, with ST912 and ST1383 being closely related members of the same clonal complex, CC912 (Table 1). The other STs (ST124, ST1365, ST1503) are not closely related to each other or to CC912, exhibiting four or more MLST allele differences.

Analysis of other isolates of the same STs (Table S1) provided strong evidence that the dual 1/2a-1/2b profile observed in ST1503 was clone specific. All other ST1503 strains identified through interrogation of the CDC and Pasteur Institute databases are serotype 1/2a but would yield the 1/2a-1/2b profile using the DS (Table S1). These strains were relatively recently isolated from human clinical samples (blood, ascites) as well as from the environment (soil) (Table S1). On the other hand, analysis of the additional strains of ST912, ST124, and ST1365 from the CDC database revealed that they would yield the serotype 1/2a/3a designation using the DS (Table S1). Thus, the dual 1/2a-1/2b profile for the ST912, ST1365, and ST124 strains in our panel (Table 1) may be unique to these strains and not characteristic of these STs, as noted above with ST1082.

It is noteworthy that while the movement of *LMOf2365_2059* into serotype 1/2a genomes appears to be recent, with the oldest strain being from 2014, this gene appears to have disseminated to multiple, unrelated serotype 1/2a STs from clinical, water, soil, and wildlife sources (Table 1; Table S1). It is also noteworthy that in the case of ST912, ST1082, and ST124, where multiple genomes of the same STs were identified upon interrogation of the databases, *LMOf2365_2059* was identified exclusively among the strains in our panel, which were from either wildlife (black bears) or surface water. It is conceivable that L. monocytogenes community composition and environmental conditions in these previously unexplored habitats may promote such HGT events or confer fitness advantages to the resulting strains.

Analysis of the *LMOf2365_2059* genomic region revealed a hypervariable mobile genetic element identified previously (36) and harboring a transposase (*orfA*) (Fig. 4). The flanking genes *lmo2025* and *lmo2028* are found in both lineages I and II, while *lmo2026* and *lmo2027* are harbored by serotype 1/2a but not 4b (Fig. 4). Analysis of this genomic region in the serotype 1/2a strains SKWL256 and SKWL233 (Doumith profiles 1/2b/3b and 1/2a-1/2b, respectively) indicated that they shared certain genes with the serotype 4b strain F2365, namely, a transposase (*orfA*), a *S*-adenosylmethionine (SAM)-dependent methyltransferase, and *LMOf2365_2059* (transcriptional regulator [*tetR*]). However, they also harbored several additional genes, namely, a glyoxalase family protein and two resolvase/integrase bin proteins (Fig. 4). Overall, similar open reading frame (ORF) organization was found in other serotype 1/2a strains with 1/2a-1/2b or 1/2b/3b profiles using the DS (data not shown). However, certain differences could be noted among strains. For instance, three ORFs harbored by SKWL233 in this region appeared absent from the genomic counterpart in SKWL256 (Fig. 4), highlighting the evolution of this mobile genetic element, which likely moved from lineage I into certain serotype 1/2a strains and diverged further in strains such as SKWL233 and SKWL256.

### Implications of horizontal gene transfer or loss of genes used as targets in molecular serotyping.

While the movement of *LMOf2365_1900*, *LMOf2365_2059*, and the *lmo0734*-*lmo0739* cassette has large impacts on L. monocytogenes isolate classifications, it is also important to consider the functional outcomes of the gain or loss of these genes. For instance, the *lmo0734*-*lmo0739* cassette is essential for d-allose metabolism in lineage II strains, and strains of lineage II can grow with d-allose as the sole carbon source, while strains of lineages I or III lack this capacity (37). However, d-allose is rare in nature, though found in certain plants, such as the African shrub Protea rubropilosa (38). It is in fact intriguing that the genes are so highly conserved in serotype 1/2a, suggesting that this cassette may have alternative functions related to virulence or other traits. The loss of this gene cassette in certain serotype 1/2a strains suggests that they may possess compensating traits (Table 1). On the other hand, strains of IVb-v1 have gained this cassette (22, 24), suggesting potential fitness advantages of such acquisition. Additional research may reveal currently unknown impacts of gain or loss of this cassette in these L. monocytogenes strains.

The serotype 4b-specific *LMOf2365_1900* encodes a trypsin-like serine protease; another L. monocytogenes serine protease, HtrA (*lmo0292*), was implicated in growth at low pH, high osmolarity, and high temperatures (39–41). *LMOf2365_1900* may contribute to the increased acid, salt, and high-temperature tolerance of serotype 4b strains compared to serogroup 1/2 (42). The potential fitness impacts of the loss of *LMOf2365_1900* in ST782 strains or the acquisition of the *LMOf2365_2059*-harboring region in ST1503 and strains of other STs remain to be elucidated. To our knowledge, ST782 is only serotype 4b clone that lacks *LMOf2365_1900*, and mechanisms potentially selecting for this loss remain to be identified. *LMOf2365_2059* is putatively annotated as a transcriptional regulator (*tetR*) and, as discussed above, is on a diverse genomic island harboring a multitude of genes, including a SAM-dependent methyltransferase and a glyoxalase family protein (Fig. 4). Methylation has numerous cellular roles, ranging from the regulation of protein-protein interactions to DNA protection (43, 44), and acquisition of this genomic island could have strong impacts on cellular fitness.

In our *Listeria* collection at North Carolina State University from the 1920s through the present, only 14 of the approximately 2,400 L. monocytogenes isolates produced the 1/2a-1/2b PCR profile, and all were subsequent to 2014, suggesting novel, recent HGT events that mobilized *LMOf2365_2059* into serotype 1/2a. This 1/2a-1/2b PCR profile is rare in current collections but may not necessarily be rare in nature. Natural environments (e.g., surface water in urban and agricultural sites, wildlife) remain undersurveilled for L. monocytogenes. As such environments become more extensively sampled, we may identify more strains with novel DS profiles or with profiles that may disagree with the actual serotype of the strains. Findings from a recent study of numerous (>1,200) L. monocytogenes strains from the Salinas River watershed (CA, USA) confirm this expectation. For instance, strains of CC842 (ST1004, ST2138, ST2139, ST2140), ST2135, and ST2137 harbor *prs* and the serotype 4b-specific *LMOf2365_1900*; they would yield a novel profile in the DS but belong to a lineage II clade based on WGS phylogeny (45).

In conclusion, HGT-mediated acquisition or loss of genes used as targets in the Doumith molecular serotyping scheme can result in novel profiles, as well as incorrect serotype designations, in certain strains. Unlike the creation of novel multiplex PCR profiles like IVb-v1 or the dual 1/2a-1/2b, which can be readily discerned, isolates of serotypes 1/2a or 4b yielding the 1/2b/3b profile using the DS can lead to incorrect serotype designations. Additional analysis using other lineage and serotype-specific PCR targets may provide more robust serotyping results (32, 46, 47). However, as with the gene targets employed in the DS, gene loss or HGT between serotypes and lineages can still result in incorrect molecular serotype designations. The growing use of WGS may alleviate this problem, and we identified no instances in our panel where classical antibody-based serotyping designations disagreed with those derived from WGS-based serotyping. However, WGS analysis of large numbers of isolates can be cost prohibitive and impractical. Thus, laboratories which rely on PCR-based molecular serotyping methods should be aware of potential outliers and incorporate additional lineage or serotype-specific primers in their multiplex PCR scheme. As indicated above, this caution is especially warranted for strains of novel genotypes and from currently underexplored sources, such as surface water and wildlife.

## MATERIALS AND METHODS

### Bacterial strains and growth conditions.

The L. monocytogenes strains used in this study are listed in Table 1. Unless otherwise specified, the strains were grown overnight at 37°C in brain heart infusion (BHI) medium (Becton, Dickinson Co, Sparks, MD, USA) or on BHI medium supplemented with 1.2% agar. Wildlife strains were isolated from feces or rectal or nasal swabs of black bears (Ursus americanus) in urban and suburban regions of the southeastern United States, as previously described (29). Strains derived from an aquatic environment in North Carolina (USA) were isolated from Rocky Branch Creek, an urban freshwater creek in Raleigh, NC, as previously described (30). All strains were preserved at −80°C with 20% glycerol.

### Serotyping of Listeria monocytogenes using agglutination and ELISA.

Somatic and flagellar *Listeria* antisera (Denka Seiken Co. Ltd., Tokyo, Japan) were used for L. monocytogenes serotyping. This agglutination-based method was performed according to the manufacturer’s instructions. For ELISA, strains were passaged three times on BHI plates containing 0.3% agar and grown at 30°C to enrich for flagellum-producing cells. For O-antigen assessment, cultures were grown in BHI broth overnight with shaking (100 to 120 rpm) at 37°C, while for H-antigen assessment, they were grown in BHI broth overnight statically at 30°C. The cultures were all centrifuged (14,000 × *g*) for 5 min. The cell pellets for the O-antigen preparations were resuspended in equal volumes of 0.2% NaCl, autoclaved for 15 min at 121°C, centrifuged again, and resuspended in 0.2% NaCl to an *A*_600_ value of 0.1 to 0.3. The cell pellets for the H-antigen preparations were resuspended in equal volumes of 4% (wt/vol) formaldehyde in 0.2% NaCl, incubated at room temperature for 30 min, centrifuged, and resuspended in 0.2% NaCl to an *A*_600_ value of 0.1 to 0.2. Cell suspensions (70 μL) were pipetted into ELISA well strips (MaxiSorp flat-bottom well strips; Nalge Nunc International, Rochester, NY) and dried overnight at 40°C in an oven. ELISA was performed using the Denka-Seika *Listeria* antiserum kit (part number 294616) and interpreted as described (48).

### Use of the Doumith scheme and *in silico* molecular serotyping.

The DS for molecular serotyping was employed as previously described and utilized five pairs of primers targeting *lmo0737*, *lmo0118*, ORF2819 (*LMOf2365_2059*), ORF2110 (*LMOf2365_1900*), and *prs* (*lmo0199*) (18). *In silico* determination of DS serotype designations used the Institut Pasteur sequence query function with the same five gene targets (25). WGS-based serotyping was achieved by utilizing core-genome MLST (cgMLST) data from the BIGSdb PasteurMLST Genome Comparator (49).

### Sequence type and clonal complex designations and analysis of specific genomic regions.

WGS-based genotyping utilized the seven-locus MLST scheme for ST and clonal complex (CC) designations using BIGSdb-*Lm*, hosted by the Institut Pasteur (25). Sequence analysis of target genes (*lmo0737*, *lmo0118*, ORF2819, ORF2110, *prs*) and investigation of their chromosomal locations were completed using the Basic Local Alignment Search Tool (BLAST) and Genome Browser functions of the PathoSystems Resource Integration Center (PATRIC) Bioinformatics Resource Center (50).

### Data availability.

Accession numbers for the WGS data for the strains used in this study are listed in Table 1. All data used in this study are available in the article or supplementary tables. The whole-genome sequence data are publicly accessible at the National Library for Biotechnology Information (NCBI).
