# Supplementary material for: Horizontal Gene Transfer and Loss of Serotype-Specific Genes in Listeria monocytogenes Can Lead to Incorrect Serotype Designations with a Commonly-Employed Molecular Serotyping Scheme
Source: Microbiol Spectr. 2022 Dec 6;11(1):e02745-22. doi: 10.1128/spectrum.02745-22 (PMC9927564; doi:10.1128/spectrum.02745-22)
Supplement: Supplemental file 1 — Table S1. Download spectrum.02745-22-s0001.pdf, PDF file, 0.1 MB [file spectrum.02745-22-s0001.pdf]

Supplementary Table S1. Additional publicly available strains of sequence types (ST) of interest. The DS serotype designations for all strains were determined in-silico with the PubMLST sequence query tool hosted by the Institut Pasteur (https://bigsdb.pasteur.fr/cgi-bin/bigsdb/bigsdb.pl?db=pubmlst_listeria_seqdef). Strains indicated with * were serotyped via agglutination or ELISA and found to be serotype 1/2a.

**Strain ID**	**Accession**	**Isolation Date**	**Source**	**ST**	**CC**	**Doumith Serotype (*in silico*)**	**WGS Serotype**
CFSAN022386	SAMN02943415	6/22/14	Organic Cucumber	1055	1055	1/2b	1/2a
PNUSAL002322	SAMN05430784	Unknown	Blood	1055	1055	1/2b	1/2a
FDA00008653	SAMN03274042	11/12/14	Sprouts	1082	1082	1/2a	1/2a
FDA00008654	SAMN03274043	11/12/14	Sprouts	1082	1082	1/2a	1/2a
FDA00008766	SAMN03299427	1/5/15	Swab	1082	1082	1/2a	1/2a
FDA00008767	SAMN03299428	1/5/15	Swab	1082	1082	1/2a	1/2a
FDA00008768	SAMN03299429	1/5/15	Swab	1082	1082	1/2a	1/2a
FDA00008770	SAMN03299431	1/5/15	Swab	1082	1082	1/2a	1/2a
FDA00008793	SAMN03332945	1/21/15	Mung bean sprout seed	1082	1082	1/2a	1/2a
FDA00008796	SAMN03340219	1/21/15	Environmental Swab	1082	1082	1/2a	1/2a
FDA00008797	SAMN03340220	1/21/15	Environmental Swab	1082	1082	1/2a	1/2a
FDA00008798	SAMN03340221	1/21/15	Environmental Swab	1082	1082	1/2a	1/2a
FDA00008799	SAMN03340222	1/21/15	Environmental Swab	1082	1082	1/2a	1/2a
FDA00008800	SAMN03340223	1/21/15	Environmental Swab	1082	1082	1/2a	1/2a
FDA00008801	SAMN03340224	1/21/15	Environmental Swab	1082	1082	1/2a	1/2a
FDA00008802	SAMN03340225	1/21/15	Environmental Swab	1082	1082	1/2a	1/2a
FDA00008803	SAMN03340226	1/21/15	Environmental Swab	1082	1082	1/2a	1/2a
FDA00008804	SAMN03340227	1/21/15	Environmental Swab	1082	1082	1/2a	1/2a
FDA00008805	SAMN03340228	1/21/15	Environmental Swab	1082	1082	1/2a	1/2a
FDA00008806	SAMN03340229	1/21/15	Environmental Swab	1082	1082	1/2a	1/2a
FDA00008807	SAMN03340230	1/21/15	Environmental Swab	1082	1082	1/2a	1/2a
FDA00008808	SAMN03340231	1/21/15	Environmental Swab	1082	1082	1/2a	1/2a
FDA00008809	SAMN03340232	1/21/15	Environmental Swab	1082	1082	1/2a	1/2a
FDA00008810	SAMN03340233	1/21/15	Environmental Swab	1082	1082	1/2a	1/2a
FDA00008811	SAMN03340234	1/21/15	Environmental Swab	1082	1082	1/2a	1/2a
FDA00008813	SAMN03340236	1/22/15	Soybean Sprouts	1082	1082	1/2a	1/2a

FDA00008814	SAMN03340237	1/22/15	Soybean Sprouts	1082	1082	1/2a	1/2a
FDA00008815	SAMN03340238	1/22/15	Soybean Sprouts	1082	1082	1/2a	1/2a
FDA00008816	SAMN03340239	1/22/15	Soybean Sprouts	1082	1082	1/2a	1/2a
PNUSAL003654	SAMN08182043	Unknown	CSF	1082	1082	1/2a	1/2a
PNUSAL000861*	SAMN02950467	7/3/14	Blood	1365	1365	1/2a	1/2a
PNUSAL004175	SAMN09779360	7/16/18	Blood	1494	1494	1/2b	1/2a
PNUSAL001678*	SAMN04009742	8/9/15	Blood, NOS	1503	1503	1/2a-1/2b	1/2a
PNUSAL002225*	SAMN05379645	5/27/16	Peritoneal fluid /ascites	1503	1503	1/2a-1/2b	1/2a
FSL L7-0609	SAMN12373159	7/7/18	Soil, MD, USA	1503	1503	1/2a-1/2b	1/2a
FSL L7-1336	SAMN11962685	10/7/18	Soil, CT, USA	1503	1503	1/2a-1/2b	1/2a
PNUSAL003100	SAMN07279076	6/5/17	Blood	1503	1503	1/2a-1/2b	1/2a
PNUSAL004594	SAMN10530944	11/18/18	Blood	1503	1503	1/2a-1/2b	1/2a
PNUSAL005637	SRS5355495	Unknown	Unknown	1503	1503	1/2a-1/2b	1/2a
PNUSAL001041	SAMN03093498	9/6/14	Blood	912	912	1/2a	1/2a
PNUSAL001947	SAMN04377792	11/21/15	Blood	912	912	1/2a	1/2a
PNUSAL002302	SAMN05413957	6/14/16	Blood	912	912	1/2a	1/2a
FDA00007952	CFSAN022374	2017	Food, TX, USA	782	2	1/2b	4b
PNUSAL002481	SRS1692697	2016	Clinical, USA	782	2	1/2b	4b
PNUSAL003505	SRS2646761	2017	Clinical, USA	782	2	1/2b	4b
PNUSAL003603	SRS2724514	2017	Clinical, USA	782	2	1/2b	4b
PNUSAL003604	SRS2724509	2017	Clinical, USA	782	2	1/2b	4b
PNUSAL003725	SRS2831038	2018	Clinical, USA	782	2	1/2b	4b
PNUSAL004434	SRS3898247	2018	Clinical, USA	782	2	1/2b	4b
PNUSAL004456	SRS3948969	2018	Clinical, USA	782	2	1/2b	4b
PNUSAL006118	SRS5544918	2019	Clinical, USA	782	2	1/2b	4b
PNUSAL012043	SRS10827173	2021	Clinical	782	2	1/2b	4b
RM50494*	CFSAN093404	2021	Water, CA, USA	782	2	1/2b	4b
AX12	SRS7311568	10/16/18	Raw potato, USA	124	124	1/2a	1/2a
PNUSAL002692	SRS1828444	Unknown	Clinical, USA	124	124	1/2a	1/2a
PNUSAL002184	SRS1452880	5/16	Clinical, USA	124	124	1/2a	1/2a
PNUSAL002151	SRS1420427	4/16	White sweet corn, USA	124	124	1/2a	1/2a
PNUSAL002066	SRS1319014	Unknown	Clinical, USA	124	124	1/2a	1/2a
PNUSAL001967	SRS1224070	12/15	Blood, NOS, USA	124	124	1/2a	1/2a
PNUSAL001882	SRS1174592	Unknown	Clinical, USA	124	124	1/2a	1/2a

PNUSAL001381	SRS918867	Unknown	Clinical, USA	124	124	1/2a	1/2a
PNUSAL001261	SRS953059	Unknown	Clinical, USA	124	124	1/2a	1/2a
PNUSAL000599	SRS574049	2/5/14	Clinical, USA	124	124	1/2a	1/2a
PNUSAL000297	SRS497131	Unknown	Clinical, USA	124	124	1/2a	1/2a
FDA959960	SRS1468363	5/9/16	Frozen mixed vegetable, CA, USA	124	124	1/2a	1/2a
FDA959953	SRS1468362	5/9/16	Frozen mixed vegetable, CA, USA	124	124	1/2a	1/2a
FDA950087-104-164	SRS1365430	3/9/16	Environmental Swab, WA, USA	124	124	1/2a	1/2a
FDA950087-103-150	SRS1365428	3/9/16	Environmental Swab, WA, USA	124	124	1/2a	1/2a
CFSAN101827	SRS5748757	2017	Environmental Swab, OR, USA	124	124	1/2a	1/2a
CFSAN093022	SRS4672710	4/9/19	Frozen peas, WA, USA	124	124	1/2a	1/2a
RM16843	CFSAN068184	1/11/2012	Water, CA, USA	124	124	1/2a	1/2a
CFSAN052647	SRS1597262	5/20/16	Pea, USA	124	124	1/2a	1/2a
CFSAN051153	SRS1473330	5/16	Frozen vegetable, CA, USA	124	124	1/2a	1/2a
CFSAN051151	SRS1473328	5/16	Frozen vegetable, CA, USA	124	124	1/2a	1/2a
CFSAN051150	SRS1473327	5/16	Frozen vegetable, CA, USA	124	124	1/2a	1/2a
CFSAN051149	SRS1473326	5/16	Frozen vegetable, CA, USA	124	124	1/2a	1/2a
CFSAN051145	SRS1473321	5/16	Frozen vegetable, CA, USA	124	124	1/2a	1/2a
CFSAN048717	SRS1401531	7/19/15	Green bean, WA, USA	124	124	1/2a	1/2a
CFSAN048716	SRS1401532	7/19/15	Green bean, WA, USA	124	124	1/2a	1/2a
CFSAN048715	SRS1401524	7/19/15	Green bean, WA, USA	124	124	1/2a	1/2a
CFSAN034726	SRS1161762	11/25/14	Onion, WA, USA	124	124	1/2a	1/2a
CFSAN034724	SRS1161753	11/25/14	Onion, WA, USA	124	124	1/2a	1/2a
Q17	SRS3801914	6/6/16	Raw potato, USA	124	124	1/2a	1/2a
Q36	SRS3801898	9/20/16	Raw potato, USA	124	124	1/2a	1/2a
S37	SRS3739832	10/7/16	Raw potato, USA	124	124	1/2a	1/2a
S43	SRS3739824	10/25/16	Raw potato, USA	124	124	1/2a	1/2a
S47	SRS3739836	11/15/16	Raw potato, USA	124	124	1/2a	1/2a
S51	SRS3739839	11/24/16	Raw potato, USA	124	124	1/2a	1/2a
